# Missed C5 vertebral brown tumor causing spinal cord compression and myelopathy: A case report and literature review

**DOI:** 10.1002/ccr3.5331

**Published:** 2022-01-28

**Authors:** Babak Mirzashahi, Farzad Vosoughi, Saied Besharaty, Sadegh Hasani Satehi

**Affiliations:** ^1^ 48439 Orthopedic Surgery Department Imam Khomeini Hospital Tehran University of Medical Sciences Tehran Iran

**Keywords:** brown tumor, case report, hyperparathyroidism, spinal cord compression

## Abstract

We aim to report a patient with vertebral brown tumor in the context of a primary hyperparathyroidism presented by shoulder pain. This is the first report of C5 brown tumor involvement in a primary hyperparathyroid patient and emphasizes the consideration of cervical vertebral evaluation in patients with persistent shoulder pain.

## Graphical abstract

This study emphasizes the consideration of cervical pathologies in patients with shoulder complaints. Cervical brown tumor is a rare entity but can be easily treated if diagnosed early.

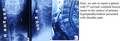

## 1 INTRODUCTION

Brown tumor is a reactive bone lesion and occurs when the bone remodeling is altered due to increased PTH level. The prevalence of brown tumor is up to 5% in primary and 13% in secondary hyperparathyroidism.^1^, ^2^ It rarely involves the spine. However, it may mimic a vertebral tumor and compress the spinal column. As it can be prevented and treated with controlling the level of parathyroid hormone, it should always be considered in the differential diagnosis of any lytic vertebral lesion. In this paper, we describe a 46‐year‐old man with a shoulder pain whose C5 vertebral body was revealed to have brown tumor. We further delineate our management and its clinical and radiographic outcomes. Our description is based on the CARE guideline checklist.

## 2 CASE REPORT

**FIGURE 1:**
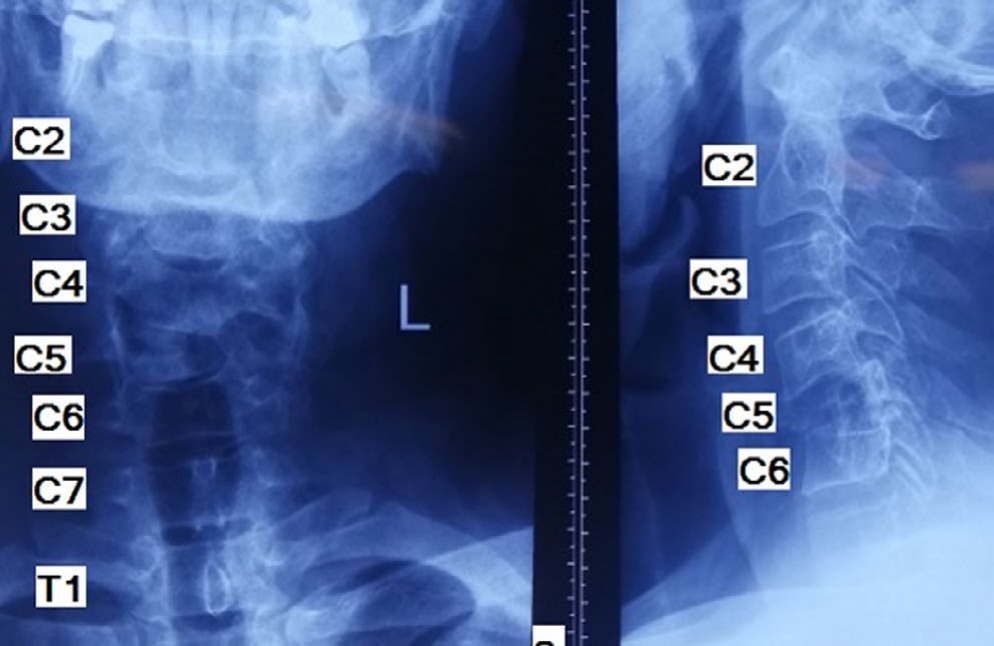
Preoperative X‐ray of the cervical spine demonstrates a lytic lesion in the C5 vertebral body causing vertebral collapse and local kyphosis

**FIGURE 2:**
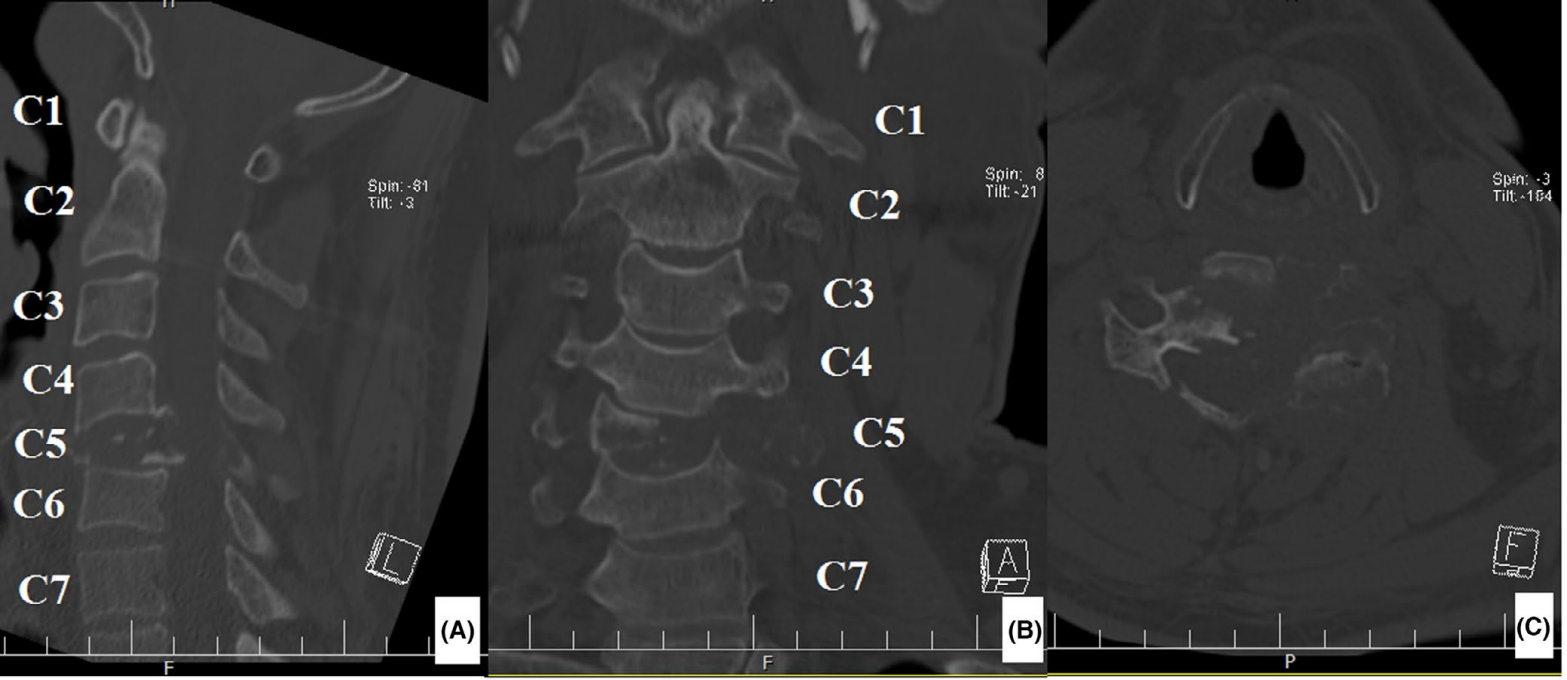
Preoperative cervical CT scan reveals a C5 lytic lesion affecting vertebral body, left sided lamina and pedicle

**FIGURE 3:**
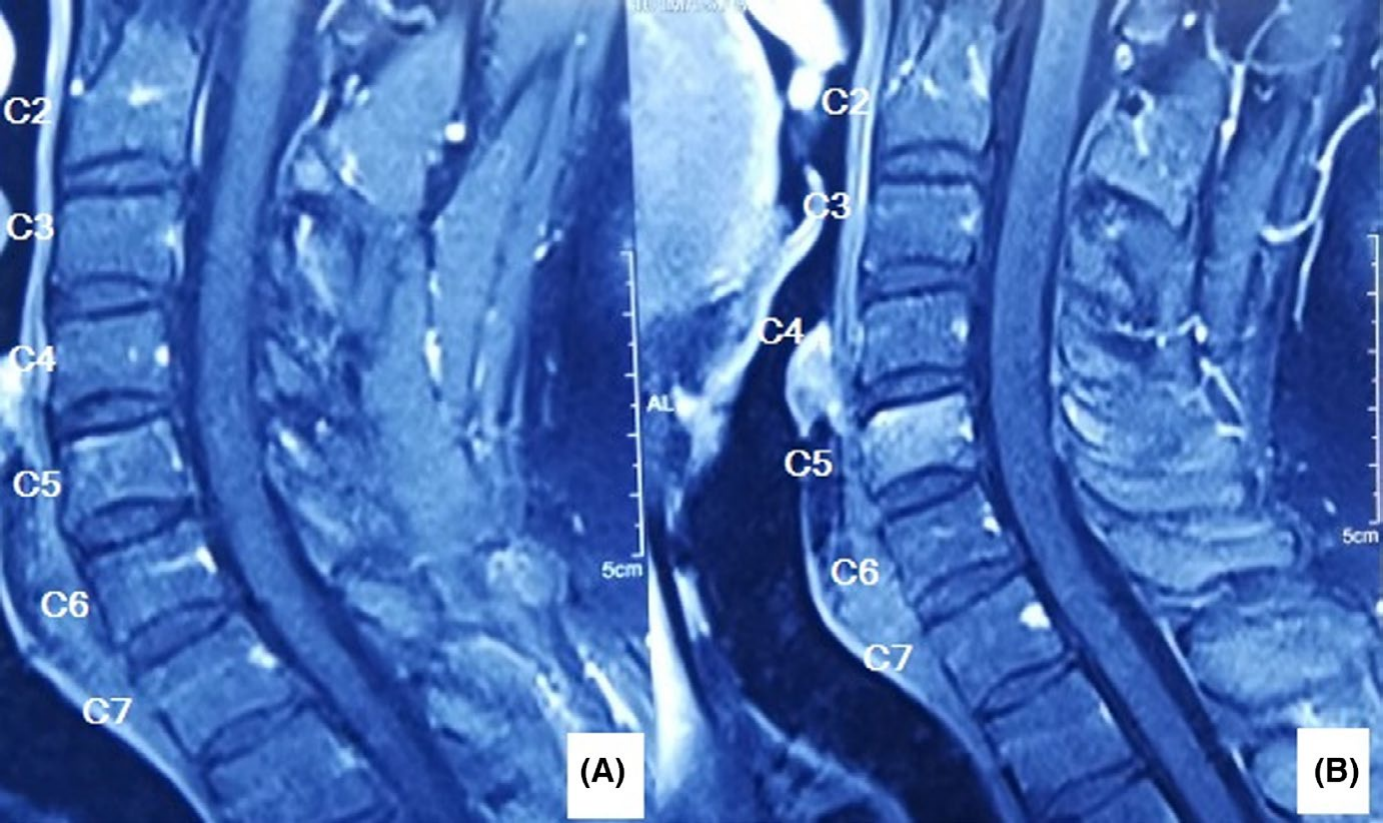
First MRI (T1) of our patient 6 months before referring to our clinic. A hyperintense signal change after IV contrast (b) is present in the C5 vertebral body

**FIGURE 4:**
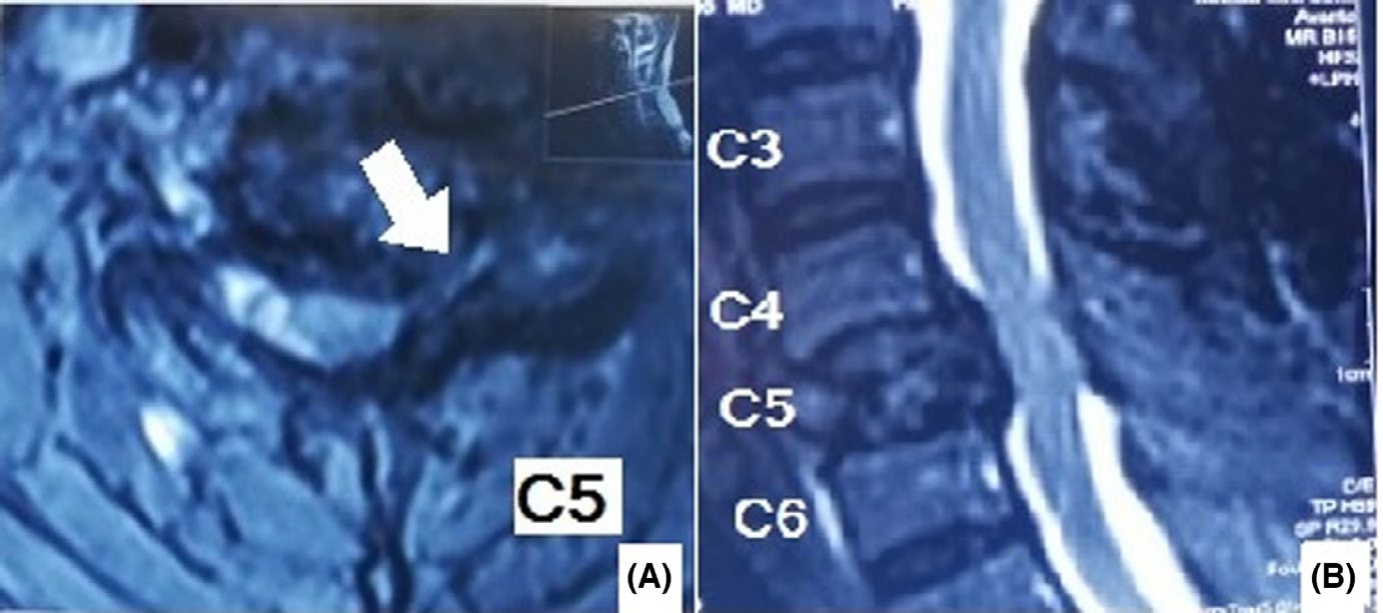
Second cervical MRI performed after exacerbation of our patient's symptoms reveals a heterogeneous lesion in the vertebral body causing vertebral collapse (A), spinal cord, and left C6 root compression (B)

A 46‐year‐old man was referred to our clinic complaining of neck pain for 8 months. Early in the course of the disease, his cervical plain X‐ray was normal. At that time, he also had a normal cervical MRI report and rheumatologic workup. Incidentally, a high level of calcium (reported level of 12.5 with a normal range of 8.7–10.7 milligram per deciliter [mg/dl]) and low level of phosphate (1.5, normal range 2.5–5 mg/dl) was detected. Upon further workup, it was determined that he had a high level of parathyroid hormone (PTH; 1285 picogram per milliliter (pg/ml), normal range 14.5–87.1 pg/ml) and serum alkaline phosphatase (ALP; 875 units per liter (U/L), normal range 80–360 U/L). He underwent a neck ultrasound that showed a lesion in favor of parathyroid adenoma (lower pole of left parathyroid lobe). His parathyroid adenoma was resected. He was recommended to undergo neck physiotherapy. Our patient experienced exacerbation of his neck pain and radiation of the pain to his left shoulder after starting physiotherapy. He was visited by a shoulder surgeon. A cervical plain X‐ray and CT scan was performed. The neck X‐ray revealed a collapse in the C5 vertebrae (Figure 1). The CT scan showed a lytic lesion in the C5 vertebrae eroding body, left pedicle, and lamina (Figure 2). As a result, he was referred to our clinic. His family and social history were unremarkable. We noticed a positive Hoffmann test. After reviewing his previous MRI retrospectively, an increased signal was noticed in the C5 vertebral body (on T1‐weighted view; Figure 3). After repeating cervical MRI, a lesion on the C5 vertebrae with a low signal on T1 and high signal on T2 was identified (Figure 4).

**FIGURE 5:**
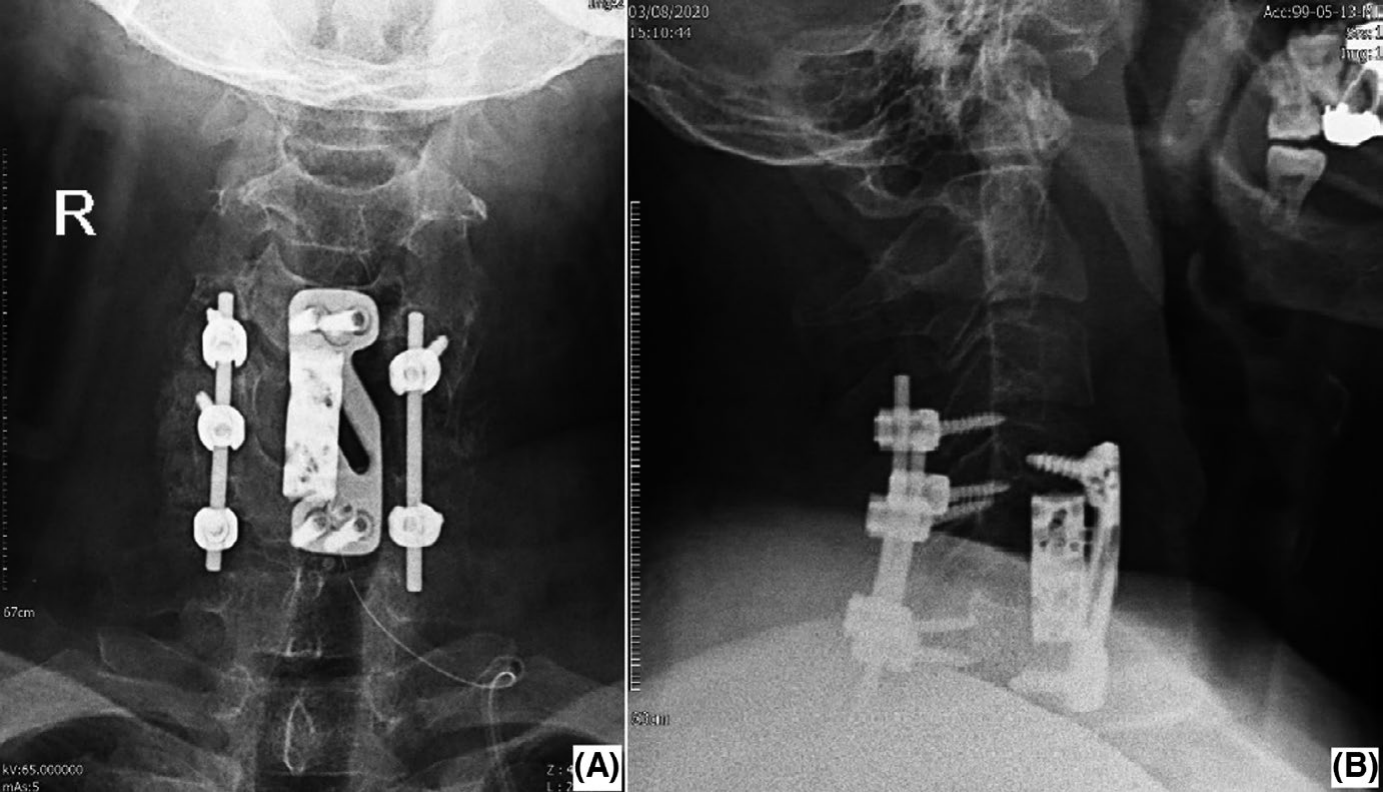
Postoperative neck X‐ray of our patient. An anterior spinal cord decompression and spinal fixation with cage and locking plate (first stage) and posterior spinal instrumentation (stage 2) were performed

**FIGURE 6:**
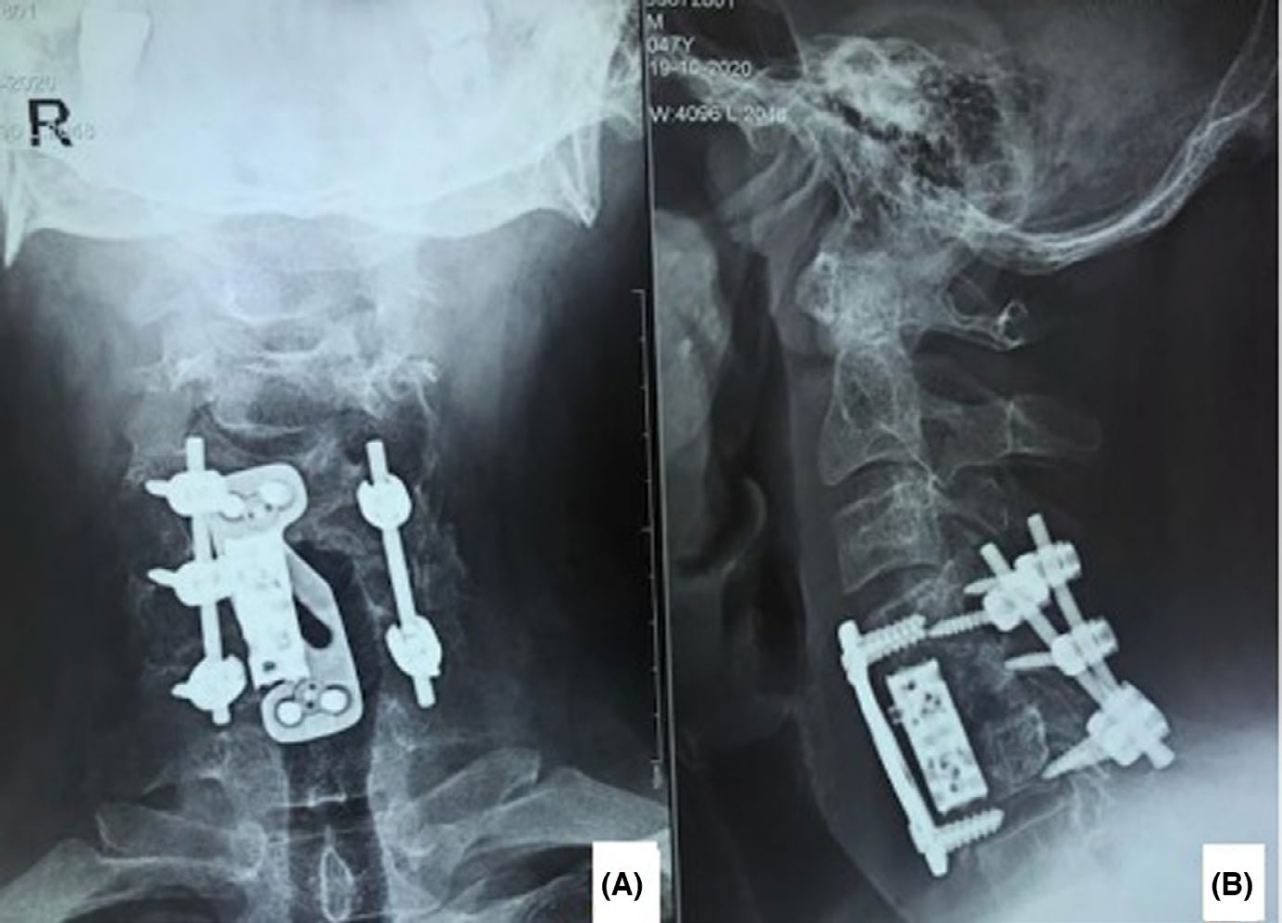
Neck X‐ray view 4 months after the spinal surgery and parathyroid adenoma resection. Evidence of C5 vertebral remineralization is present

The patient was scheduled for C5 corpectomy and cord decompression via anterior approach. During surgery, a brownish soft tissue mass within C5 vertebral body was demonstrated, and a biopsy was obtained and sent for pathology. Spinal fixation by an expandable cervical cage and cervical plate from C4 to C6 level was performed. As the C5 vertebral body involvement was extensive, posterior instrumentation with rod and lateral mass screw was done 2 days later for more rigid fixation. Due to left C5 vertebral body involvement, on its left side, no screw was used (Figure 5). The patient's symptoms were dramatically improved after the surgery, and he was recovered uneventfully. Four months postoperatively, signs of remineralization were detected on C5 vertebral body (Figure 6).

**FIGURE 7:**
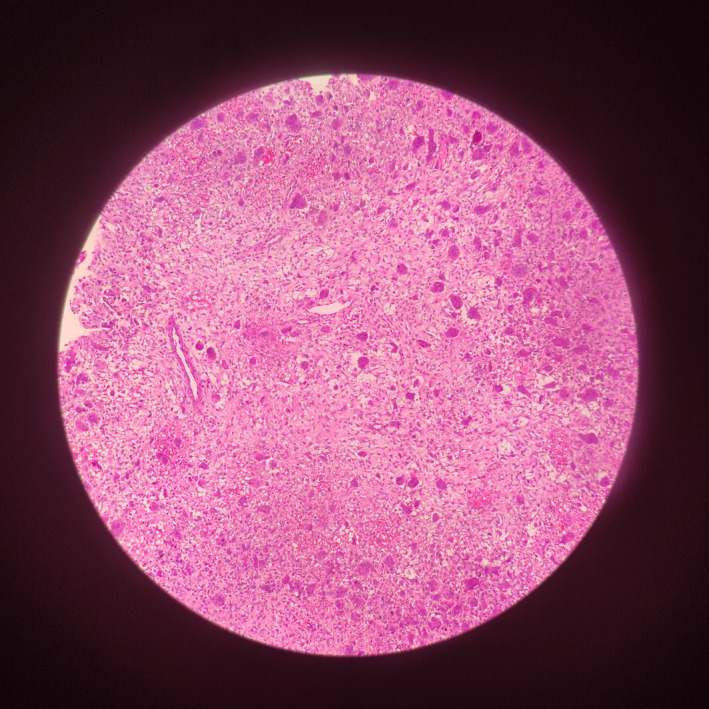
Pathology of the lesion demonstrated multinucleated giant cells in a fibrillary stroma

Scattered giant cell, peritrabecular fibrosis, and hemosiderin deposits were observed in the patient's biopsy in accordance with the diagnosis of brown tumor (Figure 7).

At the final follow‐up, 16 months after surgery, our patient was symptom‐free without any evidence of recurrence.

## 3 DISCUSSION

Brown tumor or osteoclastoma is a reactive bone lesion mainly seen in mandible sternum rib pelvis and long bones as a result of increased PTH level.^3^ Brown tumor rarely affects spinal column. To the best of our knowledge, twenty‐two cases due to primary^2^, ^4^, ^5^, ^6^, ^7^, ^8^, ^9^, ^10^, ^11^, ^12^, ^13^, ^14^, ^15^, ^16^, ^17^, ^18^, ^19^, ^20^, ^21^ and 30 cases due to secondary hyperparathyroidism^1^, ^3^, ^20^, ^22^, ^23^, ^24^, ^25^, ^26^, ^27^, ^28^, ^29^, ^30^, ^31^, ^32^, ^33^, ^34^, ^35^, ^36^, ^37^, ^38^, ^39^, ^40^, ^41^, ^42^, ^43^, ^44^, ^45^, ^46^, ^47^ were reported in the literature. Among the published reports of spinal brown tumor, it mainly affected thoracic region and rarely involves cervical area.^4^ To the extent of the authors knowledge, in the context of primary hyperparathyroidism, only three cases of cervical brown tumor have been reported in the literature (two cases involving C6 and one case affecting C2; Table 1). As far as we know, this is the first report of C5 brown tumor in a patient with primary hyperparathyroidism.

**TABLE 1:** Published English papers reporting cervical brown tumor associated with primary hyperparathyroidism

Author (year)	Country	Patient's age (sex)	Follow‐up	Level of involvement	Presenting signs/symptoms	Management	Outcome
Ashebu et.al. (2002)	Kuwait	27 y/o (female)	12 months	C6	Weakness, lethargy, painless bony swelling in both shins	Excision of the parathyroid adenoma	Symptom‐free and bony lesions healed
Alfawareh (2015)	Saudi Arabia	26 y/o (female)	24 months	C2	Axial neck pain radiating to both upper limbs, weakness of the right hand, hyperreflexia	Parathyroidectomy, halo vest	Symptoms relieved, mild atlantoaxial rotatory subluxation remained
Khalatbari (2014)	Iran	52 y/o (female)	72 months	C6	Radicular pain, weakness	Tumor resection, lateral mass screw fixation parathyroidectomy	Symptoms relieved

According to the previous papers, the spinal osteoclastoma may be associated with neurologic complaints mainly weakness, pain/paresthesia, sphincter incontinence, or pyramidal findings (upward plantar reflex and spasticity). Regarding previous reports of spinal brown tumor, the most frequent neurologic finding was sensory (pain or paresthesia) in primary hyperparathyroidism and motor weakness in secondary hyperparathyroidism. Similarly, the main complaint of our case having primary hyperparathyroidism was neck and shoulder pain. It merits attention that the lesion may also present itself with signs and symptoms of hypercalcemia due to the underlying hyperparathyroidism including the classis tetrad “kidney stone, painful bones, abdominal groans, psychic moans”, polyuria, and urolithiasis.^19^, ^36^

The lesion is associated with increased parathyroid hormone, increased level of calcium and alkaline phosphatase, and decreased level of blood phosphate. However, as previous papers reported, the level of calcium may be normal in known cases of brown tumor due to accompanied vitamin D deficiency.^4^, ^41^ This may further complicate the diagnosis of the lesion.

In plain X‐ray and CT scan, brown tumor is found as a lytic lesion. In the magnetic resonance imaging (MRI), it has low signal intensity on T1 and high/low signal intensity on T2‐weighted MRI images.^2^ It is enhanced during MRI modality with IV contrast. Osteoclastoma has a rich vascular supply and thus has a high uptake in bone scan.^36^ In accordance with previous reports, our case had a lytic, well‐defined appearance in the plain radiography and CT scan. In the cervical T1‐weighted MRI of our patient, despite previous reports, however, a hyper signal lesion and a heterogeneous lesion were noted on the first and second T1‐weighted MRI consecutively.

Occasional episodes of hemorrhage within the brown tumor and its rich vascularity give the lesion its characteristic brown appearance. Tissue biopsy of the lesion reveals peritrabecular fibrosis, hemosiderin deposits, and multinucleated giant cells. Evidence of atypia or anaplastic cells in the biopsy rules out the diagnosis of osteoclastoma.

Besides osteoclastoma, giant cell tumor and aneurismal bone cyst should be considered as a differential diagnosis in single lytic bone lesions and Paget's disease, multiple myeloma/metastasis in multiple lytic bone lesions. The giant cells have a more even distribution in giant cell tumor compared with the osteoclastoma. Also, hemosiderin deposits in the tissue biopsy and increased level of PTH are in favor of osteoclastoma. The aneurysmal bone cyst has a cystic nature with a possible fluid–fluid level in radiography. Aneurysmal bone cyst affects younger individuals (10–20 years of age).^36^ In contrast, the osteoclastoma is seen in older patients (mean age of 45.3 in primary and 39.1 in secondary hyperparathyroidism among the previous case reports) and may appear as a solid lesion in the spinal column.^4^ Regarding Paget's disease, giant cells were clustered on the surface of bone trabeculae. However, the multinucleated cells penetrates deeply through the bone and form bone tunnels in the brown tumor.^2^, ^29^

Treatment of hyperparathyroidism causes the brown tumor to regress and remineralize. However, as noted in our patient, spinal brown tumors if associated with vertebral fracture or neurologic symptoms will need an emergent spinal decompression in addition of treating the hyperparathyroidism. After spinal decompression, instrumentation and spinal fusion are performed in cases of large lesions affecting at least two vertebrae or those complicated with vertebral fracture.^4^

As brown tumor is rarely reported to involve cervical spine, this report on a complicated missed case of cervical brown tumor highlights the importance of early diagnosis and treatable nature of the lesion. However, higher level of evidence may be still needed to define its best management.

## 4 CONCLUSION

Brown tumor rarely affects the spinal column. However, as this clinical entity is preventable and treatable by diagnosing and managing hyperparathyroidism, it merits to be considered whenever a lytic vertebral lesion in encountered. This case report emphasizes the possibility of dangerous neurologic condition when a spinal brown tumor is missed and mismanaged.

## CONFLICT OF INTEREST

“The authors declare that they have no known competing financial interests or personal relationships that could have appeared to influence the work reported in this paper.”

## AUTHOR CONTRIBUTIONS

B.M conducted the surgery and supervised preparing the manuscript. F.V literature reviewed and wrote the manuscript. S.B edited the manuscript and collected the patient's data. S.H.S edited the manuscript.

## ETHICAL APPROVAL

The Ethics Committee Board of Tehran University of Medical. Sciences (TUMS) declared no ethical concern in this study.

## CONSENT

The authors are accountable for all aspects of the work in ensuring that questions related to accuracy or integrity of any part of the work are appropriately investigated and resolved. All procedures in studies involving human participants were in accordance of ethical standards of the institutional and/or national research committees and with Helsinki declaration (as revised in 2013). Written informed consent was obtained from the patient for publishing the case report and any accompanying images.

## Data Availability

Not applicable.

## References

[1] Eroglu E , Kontas ME , Kocyigit I , et al. Brown tumor of the thoracic spine presenting with paraplegia in a patient with peritoneal dialysis. CEN Case Rep. 2019;8(4):227‐232. 31089951 10.1007/s13730-019-00398-0 PMC6820650

[2] Alfawareh MD , Halawani MM , Attia WI , Almusrea KN . Brown tumor of the cervical spines: a case report with literature review. Asian Spine J. 2015;9(1):110‐120. 25705344 10.4184/asj.2015.9.1.110 PMC4330206

[3] Vandenbussche E , Schmider L , Mutschler C , Man M , Jacquot C , Augereau B . Brown tumor of the spine and progressive paraplegia in a hemodialysis patient. Spine. 2004;29(12):E251‐E255. 15187649 10.1097/01.brs.0000127187.58944.fa

[4] Khalatbari MR , Moharamzad Y . Brown tumor of the spine in patients with primary hyperparathyroidism. Spine. 2014;39(18):E1073‐E1079. 24921845 10.1097/BRS.0000000000000455

[5] Shaw MT , Davies M . Primary hyperparathyroidism presenting as spinal cord compression. Br Med J. 1968;4(5625):230‐231. 5682325 10.1136/bmj.4.5625.230 PMC1912178

[6] Shuangshoti S , Hongsaprabhas C , Chandraprasert S , Rajatapiti B . Parathyroid adenoma, brown tumor and cauda equina compression. J Med Assoc Thai. 1972;55(4):251‐258. 5022500

[7] Sundaram M , Scholz C . Primary hyperparathyroidism presenting with acute paraplegia. AJR Am J Roentgenol. 1977;128(4):674‐676. 403804 10.2214/ajr.128.4.674

[8] Ganesh A , Kurian S , John L . Complete recovery of spinal cord compression following parathyroidectomy. Postgrad Med J. 1981;57(672):652‐653. 7335567 10.1136/pgmj.57.672.652 PMC2426091

[9] Yokota N , Kuribayashi T , Nagamine M , Tanaka M , Matsukura S , Wakisaka S . Paraplegia caused by brown tumor in primary hyperparathyroidism. Case report. J Neurosurg. 1989;71(3):446‐448. 2769396 10.3171/jns.1989.71.3.0446

[10] Kashkari S , Kelly TR , Bethem D , Pepe RG . Osteitis fibrosa cystica (brown tumor) of the spine with cord compression: report of a case with needle aspiration biopsy findings. Diagn Cytopathol. 1990;6(5):349‐353. 2292220 10.1002/dc.2840060512

[11] Daras M , Georgakopoulos T , Avdelidis D , Gravani A , Tuchman AJ . Spinal cord compression in primary hyperparathyroidism. Report of a case and review of the literature. Spine. 1990;15(3):238‐240. 2191452 10.1097/00007632-199003000-00019

[12] Graziani N , Donnet A , Antipoff M , Gaborit P , Hassoun GF . Recklinhausen brown tumor of the cervical spine disclosing primary hyperparathyroidism. Neurochirurgie. 1991;37(6):394‐397. 1780018

[13] Sarda AK , Arunabh VM , Kapur M . Paraplegia due to osteitis fibrosa secondary to primary hyperparathyroidism: report of a case. Surg Today. 1993;23(11):1003‐1005. 8292854 10.1007/BF00308978

[14] Motateanu M , Déruaz JP , Fankhauser H . Spinal tumour due to primary hyperparathyroidism causing sciatica: case report. Neuroradiology. 1994;36(2):134‐136. 8183453 10.1007/BF00588079

[15] Mustonen AO , Kiuru MJ , Stahls A , Bohling T , Kivioja A , Koskinen SK . Radicular lower extremity pain as the first symptom of primary hyperparathyroidism. Skeletal Radiol. 2004;33(8):467‐472. 15221219 10.1007/s00256-004-0803-9

[16] Haddad FH , Malkawi OM , Sharbaji AA , Jbara IF , Rihani HR . Primary hyperparathyroidism. A rare cause of spinal cord compression. Saudi Med J. 2007;28(5):783‐786. 17457452

[17] Khalil PN , Heining SM , Huss R , et al. Natural history and surgical treatment of brown tumor lesions at various sites in refractory primary hyperparathyroidism. Eur J Med Res. 2007;12(5):222‐230. 17513195

[18] Altan L , Kurtoğlu Z , Yalçinkaya U , Aydinli U , Ertürk E . Brown tumor of the sacral spine in a patient with low‐back pain. Rheumatol Int. 2007;28(1):77‐81. 17569048 10.1007/s00296-007-0380-z

[19] Hoshi M , Takami M , Kajikawa M , et al. A case of multiple skeletal lesions of brown tumors, mimicking carcinoma metastases. Arch Orthop Trauma Surg. 2008;128(2):149‐154. 17354010 10.1007/s00402-007-0312-0

[20] Barlow IW , Archer IA . Brown tumor of the cervical spine. Spine. 1993;18(7):936‐937. 8316899 10.1097/00007632-199306000-00023

[21] Lee JH , Chung SM , Kim HS . Osteitis fibrosa cystica mistaken for malignant disease. Clin Exp Otorhinolaryngol. 2013;6(2):110‐113. 23799171 10.3342/ceo.2013.6.2.110 PMC3687060

[22] Ericsson M , Holm E , Ingemansson S , Lindholm T , Svendgaard NA . Secondary hyperparathyroidism combined with uremia and giant cell containing tumor of the cervical spine. A case report. Scand J Urol Nephrol. 1978;12(2):185‐187. 694445 10.3109/00365597809179990

[23] Bohlman ME , Kim YC , Eagan J , Spees EK . Brown tumor in secondary hyperparathyroidism causing acute paraplegia. Am J Med. 1986;81(3):545‐547. 3752152 10.1016/0002-9343(86)90312-8

[24] Pumar JM , Alvarez M , Perez‐Batallon A , Vidal J , Lado J , Bollar A . Brown tumor in secondary hyperparathyroidism, causing progressive paraplegia. Neuroradiology. 1990;32(4):343. 2234400 10.1007/BF00593061

[25] Fineman I , Johnson JP , Di‐Patre PL , Sandhu H . Chronic renal failure causing brown tumors and myelopathy. Case report and review of pathophysiology and treatment. J Neurosurg. 1999;90(2 Suppl):242‐246. 10199256 10.3171/spi.1999.90.2.0242

[26] Azria A , Beaudreuil J , Juquel JP , Quillard A , Bardin T . Brown tumor of the spine revealing secondary hyperparathyroidism. Report of a case. Joint Bone Spine. 2000;67(3):230‐233. 10875324

[27] Masutani K , Katafuchi R , Uenoyama K , Saito S , Fujimi S , Hirakata H . Brown tumor of the thoracic spine in a patient on long‐term hemodialysis. Clin Nephrol. 2001;55(5):419‐423. 11393391

[28] Tarrass F , Ayad A , Benjelloun M , et al. Cauda equina compression revealing brown tumor of the spine in a long‐term hemodialysis patient. Joint Bone Spine. 2007;73:748‐750. 10.1016/j.jbspin.2006.01.011 16650789

[29] Kaya RA , Cavuşoğlu H , Tanik C , et al. Spinal cord compression caused by a brown tumor at the cervicothoracic junction. Spine J. 2007;7(6):728‐732. 17998132 10.1016/j.spinee.2006.07.013

[30] Kharrat M , Turc Baron C , Djamali A , et al. Secondary hyperparathyroidism and multiple vertebral brown tumors: cure after parathyroidectomy. Nephrologie. 1997;18(4):129‐132. 9380247

[31] Mourelatus Z , Goldberg H , Sinson G , Quan D , Lavi E . Case of the month: March 1998–48 year old man with back pain and weakness. Brain Pathol. 1998;8(3):589‐590. 9669717

[32] Torres A , Mallofré C , Gómez B . Progressive paraparesia of 2 months of evolution in a 24 years‐old woman with a renal transplantation. Med Clin (Barc). 2001;116(13):510‐516. 11412611 10.1016/s0025-7753(01)71887-8

[33] Paderni S , Bandiera S , Boriani S . Vertebral localization of a brown tumor: description of a case and review of the literature. Chir Organi Mov. 2003;88(1):83‐91. 14584320

[34] Ren W , Wang X , Zhu B , Liu Z . Quiz page September 2008: progressive paraplegia in a long‐term hemodialysis patient. Brown tumor compressing the thoracic spinal column. Am J Kidney Dis. 2008;52(3):A37‐A39. 10.1053/j.ajkd.2007.12.029 18725008

[35] Pavlovic S , Valyi‐Nagy T , Profirovic J , David O . Fine‐needle aspiration of brown tumor of bone: cytologic features with radiologic and histologic correlation. Diagn Cytopathol. 2009;37(2):136‐139. 19021196 10.1002/dc.20974

[36] Mak KC , Wong YW , Luk KD . Spinal cord compression secondary to brown tumour in a patient on long‐term haemodialysis: a case report. J Orthop Surg. 2009;17(1):90‐95. 10.1177/230949900901700120 19398802

[37] Gheith O , Ammar H , Akl A , et al. Spinal compression by brown tumor in two patients with chronic kidney allograft failure on maintenance hemodialysis. Iran J Kidney Dis. 2010;4(3):256‐259. 20622318

[38] Mateo L , Massuet A , Solà M , Pérez Andrés R , Musulen E , Sánchez Torres MC . Brown tumor of the cervical spine: a case report and review of the literature. Clin Rheumatol. 2011;30(3):419‐424. 20981561 10.1007/s10067-010-1608-y

[39] Fargen KM , Lin CS , Jeung JA , Yachnis AT , Jacob RP , Velat GJ . Vertebral brown tumors causing neurologic compromise. World Neurosurg. 2013;79(1):208.e1‐208.e6. 22100293 10.1016/j.wneu.2010.02.022

[40] Resic H , Masnic F , Kukavica N , Spasovski G . Unusual clinical presentation of brown tumor in hemodialysis patients: two case reports. Int Urol Nephrol. 2011;43(2):575‐580. 20424916 10.1007/s11255-010-9738-3

[41] Mirzashahi B , Vahedian Ardakani M , Farzan A . Brown tumor of lumbar spine in chronic renal failure: a case report. Acta Med Iran. 2014;52(6):484‐487. 25130159

[42] Bertal A , Zamd M , Karkouri M , et al. Brown tumor of lumbar spine in patient with chronic renal failure. Afr J Neurol Sci. 2011;30(2).

[43] Duval‐Sabatier A , Gondouin B , Bouvier C , Bataille S , Berland Y , Brunet P . Brown tumor: still an old disease? Kidney Int. 2011;80(10):1110. 22042036 10.1038/ki.2011.290

[44] Araújo SM , Bruin VM , Nunes AS , et al. Multiple brown tumors causing spinal cord compression in association with secondary hyperparathyroidism. Int Urol Nephrol. 2013;45(3):913‐916. 22249370 10.1007/s11255-012-0123-2

[45] Tayfun H , Metin O , Hakan S , Zafer B , Vardar AF . Brown tumor as an unusual but preventable cause of spinal cord compression: case report and review of the literature. Asian J Neurosurg. 2014;9(1):40‐44. 24891890 10.4103/1793-5482.131074 PMC4038866

[46] Sánchez‐Calderón MD , Ochoa‐Cacique D , Medina Carrillo O , et al. Brown tumor of the cervical spine in a patient with secondary hyperparathyroidism: a case report. Int J Surg Case Rep. 2018;51:328‐330. 30245355 10.1016/j.ijscr.2018.09.023 PMC6153394

[47] Solmaz B , Tatarlı N , Günver F , Emre T . A thoracic vertebral brown tumor presenting with paraparesis in a patient with end‐stage renal disease. Br J Neurosurg. 2017;31(6):635‐637. 27341551 10.1080/02688697.2016.1199789

